# Outcomes of ureteroscopy (URS) for stone disease in the paediatric population: results of over 100 URS procedures from a UK tertiary centre

**DOI:** 10.1007/s00345-019-02745-3

**Published:** 2019-04-04

**Authors:** Patrick Jones, Shazna Rob, Stephen Griffin, Bhaskar K. Somani

**Affiliations:** 1 Department of Urology, University Hospital Southampton, Southampton, UK; 2 Department of Paediatric Urology, University Hospital Southampton, Southampton, UK

**Keywords:** Paediatric, Ureteroscopy, RIRS, Complications, Success, Urolithiasis

## Abstract

**Purpose:**

To report the outcomes of paediatric ureteroscopy (URS) for stone disease from a specialist endourology centre in the UK. Ureteroscopy for management of stone disease has increased worldwide and is now being done more commonly in the paediatric age group.

**Methods:**

Data were analysed retrospectively from a database maintained between April 2010 and May 2018. Consecutive patients ≤ 16 years of age undergoing semi-rigid or flexible URS for stone disease were included. Stone size and stone-free rate (SFR) were routinely assessed using an ultrasound (USS) and/or plain KUB XR. Complications were graded according to the Clavien–Dindo classification and recorded within 30 days post-procedure and readmissions within 90 days after the procedure were also captured.

**Results:**

Over the 8-year period between April 2010 and April 2018, 81 patients with a mean age of 8.8 years (range 18 months–16 years) and a male to female ratio 1:1.1 underwent 102 procedures (1.28 procedure/patient to be stone free). Of the 81 patients, 29 (35.8%) had comorbidities, with 26 (32%) having multiple comorbidities. The mean (± SEM) single and overall stone size was 9.2 mm (± 0.48, range 3–30 mm) and 11.5 mm (± 0.74, range 4–46 mm) respectively, with 22 (27.1%) having multiple stones. Thirty-five (34.7%) had stent in situ pre-operatively. The stone location was in the ureter (26.6%), lower pole (35.4%), and renal pelvis (16.5%), with 22/81(27%) having multiple stones and 21/102 (20.5%) where a ureteral access sheath (UAS) was used.

With a mean hospital stay of 1.2 days, the initial and final SFR was 73% and 99%, respectively, and 61/102 (60%) had ureteric stent placed at the end of the procedure. While there were no intra-operative complications, the readmission rate was less than 1% and there were only three early complications recorded. This included a case each of prolonged admission for pain control (grade I), urinary retention (grade II) and post-operative sepsis requiring a brief ITU admission (grade IV).

**Conclusion:**

Our study demonstrates that in appropriate setting a high stone-free rate can be achieved with minimal morbidity for paediatric patients. There is potentially a need to factor the increasing role of URS in future paediatric urolithiasis guidelines.

## Introduction

While paediatric urolithiasis remains rare, its incidence is rising [1, 2]. Furthermore, for those individuals affected by stone disease, recurrence can occur in up to 50% of cases [3, 4]. Therefore, the need to establish and develop effective and safe surgical treatments is paramount. The evolution of minimally invasive surgical interventions such as ureteroscopy (URS) has been central to this process [5, 6]. Moreover, advancements related to optic systems, equipment and surgeon expertise have propelled URS as the intervention of choice for many paediatric stone cases [7, 8, 9]. Accordingly, its uptake and dissemination has seen a marked rise across the world since the first description of its application in the paediatric setting in 1988 by Ritchey et al. [10, 11, 12].

While the uptake of URS has increased, questions still remain on the outcomes pertaining to the stone-free rate (SFR) and complications of this procedure. We hypothesise that paediatric URS achieves excellent SFR with minimal intra or post-operative complications when done using a standardised protocol-based approach. This study aims to report the outcomes of paediatric URS cases from a specialist endourology centre in the UK.

## Methods

Data were analysed retrospectively from a database maintained between April 2010 and May 2018. Cross-checking and analysis were done using the electronic operative notes, laboratory systems, hospital discharge records and correspondence. Consecutive patients under 16 years of age undergoing URS (rigid or flexible) were included. We receive paediatric urolithiasis referrals from a geographic area ranging up to 350 km including complex cases from other units. Stone size and stone-free rate (SFR) were routinely assessed using an ultrasound (USS) and/or plain KUB XR. All patients routinely had urine culture and if positive this was treated prior to URS. Each case was discussed at a dedicated stone multidisciplinary team (MDT) meeting attended by an adult endourologist, paediatric urologist, interventional radiologist and biochemical pathologist.

All procedures were performed under general anaesthetic. Semi-rigid and flexible URS was performed using Richard Wolf 4.5–6 F and Storz FlexX2 7.5 F, respectively. Stones were treated with Holmium YAG laser [Versa Pulse Holmium Powersuite 100 W or 20 W Lumenis (UK) Ltd., Elstree, UK] using a 272-μm laser fibre (Lumenis, Inc.) and/or basket extraction. In select cases, especially for larger stones, an access sheath (9.5 F/11.5 F Cook Flexor sheath) was employed to augment drainage, collection of fragments and maintain a lower intra-renal pressure for longer cases [13]. When possible, an attempt was made to remove a stone fragment for stone analysis using a Cook NGage^**®**^ nitinol stone extractor (Cook Medical, USA) and send for biochemical analysis. The full details of the procedural steps have been published previously [14, 15].

A 4.7 F ureteric stent (Cook Medical, USA) or an overnight ureteral catheter was selectively inserted and was removed 6–8 weeks post-operatively under general anaesthesia. Stone-free status was determined both endoscopically at the end of the procedure and radiologically (USS) at 3 months follow-up prior to outpatient review. Stone-free rate (SFR) was defined as fragments < 2 mm in size [16–18]. Complications were graded according to the Clavien–Dindo classification and recorded within 30 days post-procedure [19]. Readmissions within 90 days after the operation were also captured. To be characterised as an episode of stone recurrence, previous stone clearance must have been confirmed radiologically and at least 6 months passed since the previous operation.

## Results

Eighty-one patients with a mean age of 8.8 years (range 18 months–16 years) and a male to female ratio 1:1.1 underwent 102 procedures (1.28 procedure/patient to be stone free) (Table 1). However, including the pre-operative stent insertions (*n* = 35), the mean number of procedures was 1.7 procedures/patient. Of these procedures, seven were diagnostic URS where the stone had either passed (*n* = 3) or it revealed papillary calcification (*n* = 3) or a polyp (*n* = 1). With the exception of three cases, the procedures were all carried out in the elective setting. Seven patients developed recurrent stone disease requiring repeat surgical intervention. Bilateral synchronous URS was performed in two cases.

**Table 1:** Outcomes of ureteroscopy for stone disease in paediatric age group (ICU—intensive care unit)

Summary table	
Demographics	
Total number of patients	81
Total number of ureteroscopy procedures	102
Male: female ratio	1:1.1
Mean age	8.8 years (range 1.5–16 years)
Stone attributes	
Mean single stone size	9.2 mm (range 3–30 mm)
Mean cumulative stone size	11.5 mm (range 4–46 mm)
Stone location	Upper pole—8.8% Mid pole—12.7% Lower pole—35.4% Renal pelvis—16.5% Upper ureter—5.1% Mid ureter—6.3% Distal ureter—15.2%
Multiple stones	22/81 (27%)
Pre-operative positive urine culture	15/102 (14%)
Pre-operative stent	35/102 (34%)
Post-operative stent	61/102 (60%)
Access sheath use	21/102 (20.5%)
Results	
Initial stone free rate	73%
Final stone free rate	99%
Number of URS procedures/patient for being stone free (excluding 7 diagnostic URS cases)	1.28 (range 1–3) 54—1 procedure 19—2 procedures 1—3 procedures
Stone analysis available in 52/81 (61.7%) patients	
Calcium oxalate	13
Calcium phosphate	12
Magnesium ammonium phosphate (MAP)	10
Calcium oxalate and calcium phosphate	8
Calcium phosphate and MAP	5
Cystine	3
Uric acid	1
Length of stay	1.2 days (range 1–5 days)
Complication rate	3%
Re-admission rate	0.9%

Of the 81 patients, 29 (35.8%) had comorbidities including epilepsy (*n* = 7, 8.6%) and cerebral palsy (*n* = 6, 7.4%), with 26 (32%) patients having multiple comorbidities. 23/81 (28.3%) patients had an underlying metabolic abnormality, the commonest being hypercalciuria (*n* = 16, 20%). Similarly, anatomical abnormality was present in 27/81 (33%) cases and included horseshoe kidney, mega-ureter, transplant kidney and PUJ obstruction.

Mean single stone size was 9.2 mm (range 3–30 mm) with a cumulative stone size of 11.5 mm (range 4–46 mm) and 22 (27.1%) having multiple stones. Thirty-five patients (34.7%) had stent in situ pre-operatively. Only two (1.9%) cases were initially unsuccessful due to ureteric narrowing, but after stent placement, at second operation, both these cases were successful. The index stone location was in the upper pole 8.8%, mid pole 12.7%, lower pole 35.4%, renal pelvis 16.5%, upper ureter 5.1%, mid ureter 6.3% and lower ureter 15.2%. 21/102 (20.5%) procedures utilised ureteric access sheath (Table 1). 15/102 (14.7%) of cases had positive urine culture pre-operatively, of which *Proteus* and *E. coli* represented the commonest pathogens. These were managed with the appropriate antibiotics as per sensitivities and microbiology advice, and upon confirmation of sterile urine planned URS was carried out.

The SFR after the first URS was 73% (*n* = 54). Of the remaining patients, 19 needed a second procedure and 1 needed a third procedure to be stone free. Therefore, the final SFR was 99%, which needed 1.28 procedures/patient to be stone free. Sixty percent (61/102) had ureteric stent placed at the end of the procedure. The mean length of stay was 1.2 days (range 1–5). Results of stone analysis were available in 52/81 (61.7%) patients. The stone analysis is shown in Table 1.

Only one patient was not rendered stone free, wherein a 6 mm lower pole stone was managed with surveillance as per patient/family wishes. The mean number of procedures to achieve stone-free status was 1.28 (range 1–3). While there were no intra-operative complications, the readmission rate was less than 1% and there were only three early complications recorded. This included a case each of prolonged admission for pain control (grade I), urinary retention needing a urethral catheter (grade II) and post-operative sepsis requiring a brief ITU admission (grade IV).

## Discussion

### Key findings

Our paper highlights the safety and efficacy of ureteroscopy for stone disease in the paediatric population. With our ‘twin surgeon’ approach, excellent SFR was achieved with no intra-operative complication and a very small risk of post-procedural complication [15]. Given that more than half of these patients had multiple comorbidities or metabolic or anatomical abnormality, specialist endourology centres with dedicated paediatric urology team could be a way forward to managing these complex paediatric stone patients. Larger stones may need a staged procedure for achieving a stone-free status.

### Meaning of our study and approaches to minimising complications

Through our approach by having a twin surgeon model of managing these patients, by teaming a paediatric surgeon/urologist who is more experienced in looking after these paediatric patients and an adult endourologist high-volume surgeon who is doing these procedures routinely, the outcomes are much better than those reported in the literature [15]. Similarly, following the steps of the procedure where a cystoscopy and safety guidewire placement is followed by a semi-rigid ureteroscopy over an access guidewire, and a flexible ureteroscopy then done with or without an access sheath, the procedure seems to decrease the intra-operative complication rates [14, 15, 20]. Furthermore, in patients where a primary URS is not possible, placement of a ureteric stent and delayed URS is a safe strategy, and this threshold is lower for patients under 6 years of age. For larger stones, a planned staged URS procedure might be an alternative to the PCNL procedure. Similarly, since most patients would need a further general anaesthetic for stent removal, we recommend a second look at the time of stent removal and removing any remaining fragments as opposed to pushing the boundaries during the first ureteroscopic procedure [14].

### Role of SWL and PCNL

The 2016 European paediatric stone guidelines put forward SWL as the first line intervention for urolithiasis with a number of exceptions [21]. For distal ureteric stones, URS is the treatment of choice, and for renal stones > 2 cm, lower pole stones > 1 cm and staghorn stones, PCNL is preferred. However, the disadvantages of SWL include the need for general anaesthetic for paediatric patients and can necessitate multiple sessions to achieve stone-free status. The SFRs reported for SWL in current literature are also inferior to that achieved by URS [22, 23].

A recent study by Pietropaolo et al. reveals that over the past 20 years, while SWL has been the most common treatment modality, in more recent years the dissemination of URS has risen markedly [10]. Rob et al. reported that there was no significant difference in the complication rates of URS (both major and minor) between medium and high-volume paediatric centres [24]. A recent meta-analysis by Geraghty et al. also found the overall cost of URS procedure to be lower than SWL ($2801) compared to SWL ($3627) ($2801 vs. $3627, *p* = 0.03) [25].

The role of PCNL has also expanded for both adult and paediatric stone disease [26–30]. Saad et al. reported on their randomised trial of URS versus PCNL in 38 children under 16 years old with large renal stones (> 2 cm) [31]. Although SFR was significantly lower in the URS group (71% vs 95.5%, *p* = 0.046), radiation time and hospital stay were longer with PCNL (*p* < 0.001). In addition to this, PCNL was associated with more complications (*p* = 0.018) and a higher blood transfusion rate (*p* = 0.015). These can however be minimised with advancements related to the emergence of miniaturised PCNL techniques [32]. Use of PCNL access allows for lower treatment failure; however, the use of miniaturised techniques can reduce visual quality [33]. Further randomised trials comparing URS and miniaturised PCNL are warranted.

### Role of ureteroscopy (URS)

While URS has been established as a safe procedure for paediatric patients, the failure rate and complications are higher in patients < 6 years [34]. With a lower morbidity compared to PCNL and a higher SFR compared to SWL, it seems that URS is evenly poised between these two interventions and offers a balanced solution for paediatric stones. With further digitisation of technology, reduction in scope size and improved laser fragmentation, it seems that the uptake of URS is going to increase for paediatric patients. Similarly, its role in complex patient groups or bilateral urolithiasis is likely to continue to evolve [35, 36]. Recent evidence shows that outcomes for bilateral URS procedures are at least equivalent to staged procedures, minimising cost and hospital stay [36]. Perhaps, the paediatric urolithiasis guidelines need to address the role of access sheath, and ureteroscopy for large stones and lower pole stones.

## Strengths and limitations

This represents one of the largest observational studies on paediatric URS and reflects analysis of outcomes collected from our database. The lack of randomisation increases the risk of selection bias. We went through all correspondences, results and hospital documentation to capture the complications and readmissions, but our study has the limitations of being a retrospective study. Overall, 34% patients needed a pre-operative stent requiring a prior general anaesthetic procedure. We did not formally assess the intra-renal pressure and there is a lack of long-term follow-up data. However, all procedures were done by an endourologist with a paediatric urologist demonstrating the efficacy and safety of this twin surgeon model approach.

## Conclusion

Our study demonstrates that in an appropriate setting, a high stone-free rate can be achieved with minimal morbidity for paediatric patients. There is potentially a need to consider the increasing role of URS in future paediatric urolithiasis guidelines.
